# Survey of European neurosurgeons’ management of unruptured intracranial aneurysms: inconsistent practice and organization

**DOI:** 10.1007/s00701-020-04539-8

**Published:** 2020-09-01

**Authors:** Torbjørn Øygard Skodvin, Roar Kloster, Wilhelm Sorteberg, Jørgen Gjernes Isaksen

**Affiliations:** 1 Faculty of Health, UiT The Arctic University of Norway, Tromsø, Norway; 2 Department of Neurosurgery, University Hospital of Northern Norway, Tromsø, Norway; 3 Hospital of Southern Norway, Kristiansand, Norway; 4 Department of Neurosurgery, Oslo University Hospital Rikshospitalet, Oslo, Norway

**Keywords:** Intracranial aneurysms, Subarachnoid haemorrhage, Decision-making, Guidelines, Management routines

## Abstract

**Background:**

The discovery of an unruptured intracranial aneurysm creates a dilemma between observation and treatment. Neurosurgeons’ routines for risk assessment and treatment decision-making are unknown. The position of evidence-based medicine in European neurosurgery is considered to be weak, high-grade guidelines do not exist and variations between institutions are probable. We aimed to explore European neurosurgeons’ management routines for newly discovered unruptured intracranial aneurysms.

**Methods:**

In cooperation with the European Association of Neurosurgical Societies (EANS), we conducted an online, cross-sectional survey of 420 European neurosurgeons during Spring/Summer 2016 (1533 non-Norwegians invited through the EANS, and 16 Norwegians invited through heads of departments because of the need for additional information for a separate study). We asked about demographic variables, routines for management and risk assessment of newly discovered unruptured intracranial aneurysms and presented a case. We collected information about gross domestic product (GDP) per capita from the International Monetary Fund.

**Results:**

The response rate to the invite from the EANS was 26%, with respondents from 47 countries. More than half of the respondents (*n* = 226 [54%]) reported that their department treated less than 25 unruptured aneurysms yearly. Forty percent said their department used aneurysm size cut-off to guide treatment decisions, with a mean size of 6 mm. Presented with a case, respondents from countries with a lower GDP per capita recommended intervention more often than respondents from higher-income countries. Vascular neurosurgeons more commonly recommended observation.

**Conclusion:**

The answers to this self-reported survey indicate that many centers have a treatment volume lower than recommended by international guidelines, and that there are socioeconomic differences in care. Better documentation of treatment and outcome, for example with clinical quality registries, is needed to drive improvements of care.

**Electronic supplementary material:**

The online version of this article (10.1007/s00701-020-04539-8) contains supplementary material, which is available to authorized users.

## Introduction

Extended use of cerebral imaging has led to growing numbers of incidentally discovered unruptured intracranial aneurysms [1]. This creates a dilemma as the future rupture risk of an aneurysm must be weighed against its treatment risks. Endovascular and surgical techniques may thus secure an aneurysm from future rupture, but this treatment contains a certain calculated risk.

High-grade guidelines do not exist for treatment decisions regarding unruptured intracranial aneurysms [2–4]. Risk assessment tools such as PHASES [5] (population, hypertension, age, size of aneurysm, earlier SAH from another aneurysm, and site of aneurysm) score and UIATS [2] (unruptured intracranial aneurysm treatment score) have been developed but have been subjected to criticism. Consequently, decision-making and advising a patient with a newly discovered intracranial aneurysm largely depend on the clinicians managing the specific patient. Which aneurysm and patient factors that are considered most important for the rupture risk of aneurysms hence differ substantially even between highly informed individuals [6]. Furthermore, the position of evidence-based medicine in European neurosurgery has been considered to be weak, with local “house rules” taking precedence over clinical evidence [7]. These factors combined provide fertile ground for variations in treatment between institutions. How neurosurgeons assess risk and make treatment decisions regarding unruptured intracranial aneurysms is largely unknown.

The aim of this study was to investigate European neurosurgeons’ management of newly discovered unruptured intracranial aneurysms. We further sought to explore possible differences between treatment volume, geographical regions and income levels. To this end, a self-reporting questionnaire was developed and made available to the members of the European Association of Neurological Surgeons (EANS) through an online survey tool.

## Methods and materials

This cross-sectional study is a cooperation between the four neurosurgical centers treating all intracranial aneurysms in Norway, and the EANS.

### Questionnaire development

Experienced vascular neurosurgeons, epidemiologists with experience from international surveys, and a review of relevant literature formed the basis for development of a 21-item English-language questionnaire exploring management routines in newly discovered unruptured intracranial aneurysms. The questionnaire had a structured format with closed-ended questions to maximize response rate and ease of answering [8].

**Fig. 1:**
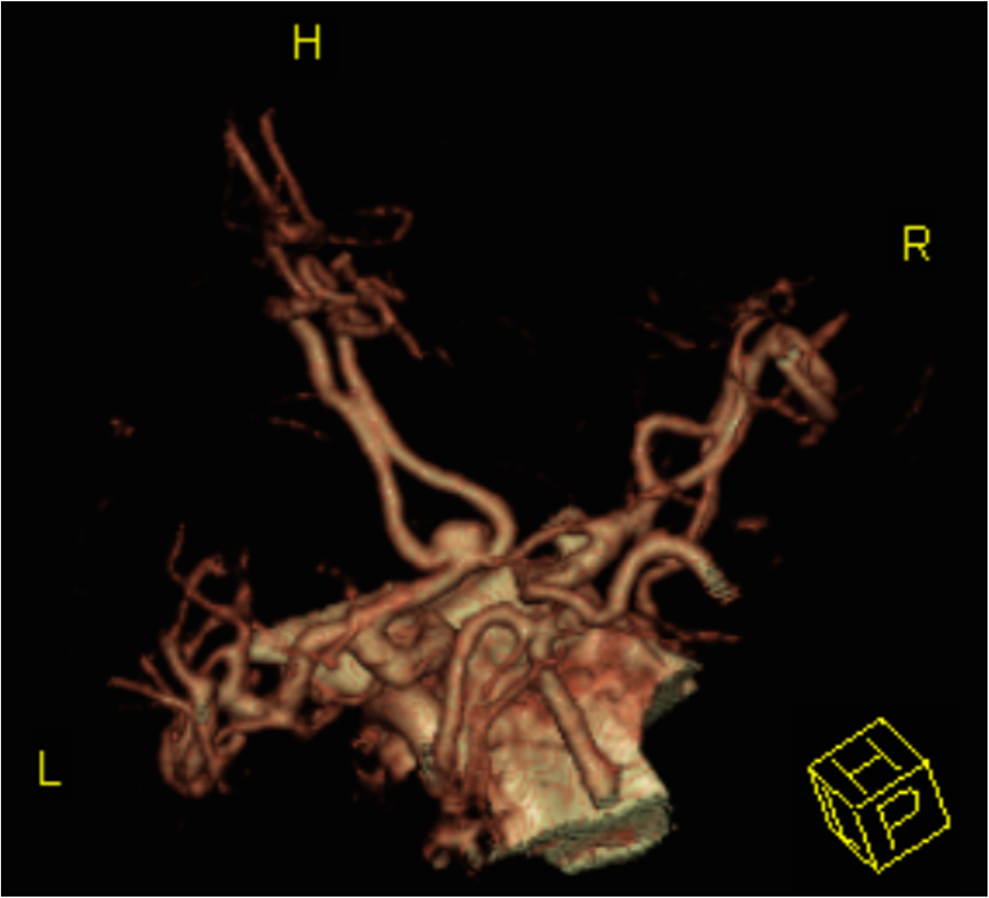
Computed tomography angiography image accompanying a case in the questionnaire, with the following text: “In your outpatient clinic, a 65-year old male presents with an anterior communicating artery (ACOM) aneurysm. The aneurysm has been incidentally discovered, and has a maximal diameter of 5 mm. The aneurysm wall is somewhat inhomogenous. The neuroradiologist informs you that endovascular treatment is possible. The patient is diagnosed with hypertension, but does not smoke and is otherwise healthy. He is willing to undergo prophylactic treatment, but is not particularly anxious about the risk of rupture in the case of abstaining from prophylactic procedures. Above is a CTA image of the aneurysm and surrounding vasculature.”

The first section examined the respondents’ age, gender, country, type of practice, position and role in decision-making with regard to aneurysm patients and the volume of aneurysm patients in their department. The respondents were then asked about the routines for discovery, risk assessment, and decision-making with regard to newly discovered unruptured intracranial aneurysms at their respective departments. The middle section contained Likert-style rating questions for the respondents to appraise the importance of different factors for decisions with regard to risk of aneurysm rupture and treatment. The final section contained a case, as shown in Fig. 1. The aim of the case was not to settle a “right” answer but rather to elicit factors influencing the respondents’ answers. The complete questionnaire is available as Electronic supplementary material 1.

### Questionnaire administration

The EANS sent an e-mail during the Spring of 2016 to all of its non-Norwegian members (*n* = 1533) inviting them to participate in the survey. The survey was administered through an online survey tool (SurveyMonkey.com, Palo Alto, CA, USA).

The invitation e-mail informed about the aim of the survey and provided a link where the receiver could easily indicate whether he or she wanted to participate or not. Three and six weeks after the first invitation, a reminder e-mail was sent to all non-respondents who had not declined to participate.

As additional information for a separate study was collected from Norwegian neurosurgeons, we needed to collect their responses outside of the online tool. Therefore, the heads of the four Norwegian neurosurgical departments received printed versions of the survey, which they distributed to doctors at their respective departments during Spring/Summer 2016. The printed versions had prepaid return envelopes enclosed.

The participants were assured that all answers would be handled confidentially, and that they could not be traced back to the individual respondent. Respondents could skip any question. In accordance with Norwegian law, the study is exempt from consideration by the committee for medical research ethics as it involves no patients and/or interventions. The study is reported according to the Strengthening the Reporting of Observational Studies in Epidemiology (STROBE) statement for observational studies [9].

### Statistical analysis

The data were analyzed with Stata for Mac (version 15; StataCorp LP, TX, USA). We summarized categorical variables as percentages. Categorical variables were compared using chi squared test. Associations between continuous variables were explored using linear regression, and between nominal and continuous variables using multinomial regression. A *p* value of < 0.05 was assumed statistically significant. Missing items were excluded from analysis.

The practice setting was dichotomized as university hospital or other. The respondents’ countries were grouped into geographical regions according to United Nations geoscheme for Europe [10]: *Northern* (Iceland, Denmark, Estonia, Faroe Islands, Finland, Guernsey, Iceland, Ireland, Isle of Man, Jersey, Latvia, Lithuania, Norway, Sark, Svalbard and Jan Mayen, Sweden, UK), *Western* (Austria, Belgium, France, Germany, Liechtenstein, Luxembourg, Monaco, Netherlands, Switzerland), *Southern* (Albania, Andorra, Bosnia and Herzegovina, Croatia, Gibraltar, Greece, Italy, Macedonia, Malta, Montenegro, Portugal, San Marino, Serbia, Slovenia, Spain, Vatican City) and *Eastern* regions (Belarus, Bulgaria, Czech Republic, Hungary, Poland, Moldova, Romania, Russia, Slovakia, Ukraine), as well as *Others* (Turkey, Israel). Gross domestic product (GDP) per capita for 2016 for each country was collected from the International Monetary Fund [11].

In the questionnaire, respondents were asked to provide numbers of unruptured aneurysms evaluated and treated each month at their department. In “Results” section, we present these answers as numbers per year for easier comparison with previous studies and guidelines.

## Results

The EANS invited its 1533 members, of whom 404 (26%) responded. In addition, there were 16 Norwegian respondents. The respondents resided in 47 different countries.

### Characteristics of participants and responders’ department

Table 1 describes the self-reported characteristics of respondents and respondents’ department. The majority was male (*n* = 369 [88%]), worked at a university/teaching hospital (*n* = 314 [75%]), and the mean age was 41 years (standard deviation, ± 10).

**Table 1:** Characteristics of respondents and respondents’ departments

Variable	No. of respondents (%)
All	420 (100)
Individual responders	
Male	369 (88)
Age, mean (±SD)	41 (10)
Region	
Western Europe	144 (34)
Northern Europe	65 (16)
Southern Europe	116 (28)
Eastern Europe	65 (16)
Other	30 (7)
Practice setting	
University/teaching hospital	314 (75)
General/regional hospital	74 (18)
Private clinic/hospital	24 (6)
Other	8 (2)
Position and role	
Head of department	72 (17)
Consultant—vascular neurosurgeon	64 (15)
Consultant—general neurosurgeon or other subspecialty	144 (34)
One of the surgeons responsible for final treatment decisions about aneurysms	65 (16)
Part of a vascular team responsible for final treatment decisions about aneurysms	89 (21)
Other^*^	107 (26)
Procedures that the responder performs	
Surgical clipping	287 (68)
Endovascular coil	37 (9)
Endovascular stent/flow diversion	24 (6)
Bypass operation	52 (12)
Other	67 (16)
Responders’ department	
Procedures performed at the department	
Surgical clipping	372 (89)
Endovascular coil	364 (87)
Endovascular stent/flow diversion	309 (74)
Bypass operation	115 (27)
Other	26 (6)
No. of patients with a newly discovered unruptured aneurysm *evaluated* for treatment in a typical month	
< 1(< 12 per year)	81 (19)
2–4(12–48 per year)	170 (41)
5–15(49-180 per year)	141 (34)
16–30(181–360 per year)	21 (5)
> 30(> 360 per year)	7 (2)
No. of patients with an unruptured aneurysm *treated* surgically/endovascularly in a typical month	
< 1(< 12 per year)	97 (23)
1–2(12–24 per year)	129 (31)
3–5(25–60 per year)	118 (28)
6–10(61–120 per year)	59 (14)
> 10(> 120 per year)	17 (4)
The role mainly/always responsible for final treatment decisions	
Vascular team	294 (78)
Individual neurosurgeon	143 (38)
Individual neuroradiologist	76 (20)
Individual neurologist	9 (2)
No. of neurosurgeons involved in different phases of unruptured intracranial aneurysms evaluations at department, median (IQR)	
Rupture risk assessment	3 (2–4)
Final treatment decisions	3 (2–4)
Surgical/endovascular treatment	3 (2–4)
Normal waiting times, median no. of weeks (IQR)	
From reception of referral and to patient is seen in outpatient clinic	2 (1–4)
From the patient is seen in outpatient clinic to final treatment	4 (2–6)
Does the department use aneurysm size cut-off to guide treatment decisions?	166 (40)
Cut-off size for departments that used such, mean (±SD)	6.0 (2.4)

Respondents could skip any question. The reported percentages are calculated from the number of respondents for each question

*IQR* interquartile range, *SD* standard deviation.

^*^Eighty-seven of these specified that they were residents or similar terms

More than half of the respondents (*n* = 226 [54%]) reported that their department treated less than 25 unruptured aneurysms yearly. In Southern and Eastern Europe, this treatment volume was reported by 66% and 63%, respectively; 43% in Northern and 40% in Western Europe (data shown in Electronic supplementary material 2).

According to tertiles of GDP per capita, 142 of the 415 respondents (34%) practiced in countries that belonged to the lowest tertile, 191 (46%) to the middle and 82 (20%) to the highest tertile. Electronic supplementary material 2 provides details with regard to means and ranges of GDP per capita for the tertiles and for the European geographical regions.

### Managing routines

Seventy-eight percent of respondents said vascular teams “mainly” or “always” make the final treatment decisions for patients with unruptured intracranial aneurysms. Stratified by GDP per capita tertiles, 62% of responders in the lowest tertile said vascular teams “mainly” or “always” make these decisions, in contrast to 86% and 85% for the middle and highest tertile, respectively.

The image modalities “mainly” or “always” used to guide treatment decisions ranged from 40% for magnetic resonance angiography and 45% for 2-dimensional digital subtraction angiography to 65% for both computed tomography angiography and 3-dimensional digital subtraction angiography.

Tables displaying the relationships between choice of image modality, GDP per capita, and European geographical regions are provided in Electronic supplementary material 2.

### Risk assessment

**Fig. 2:**
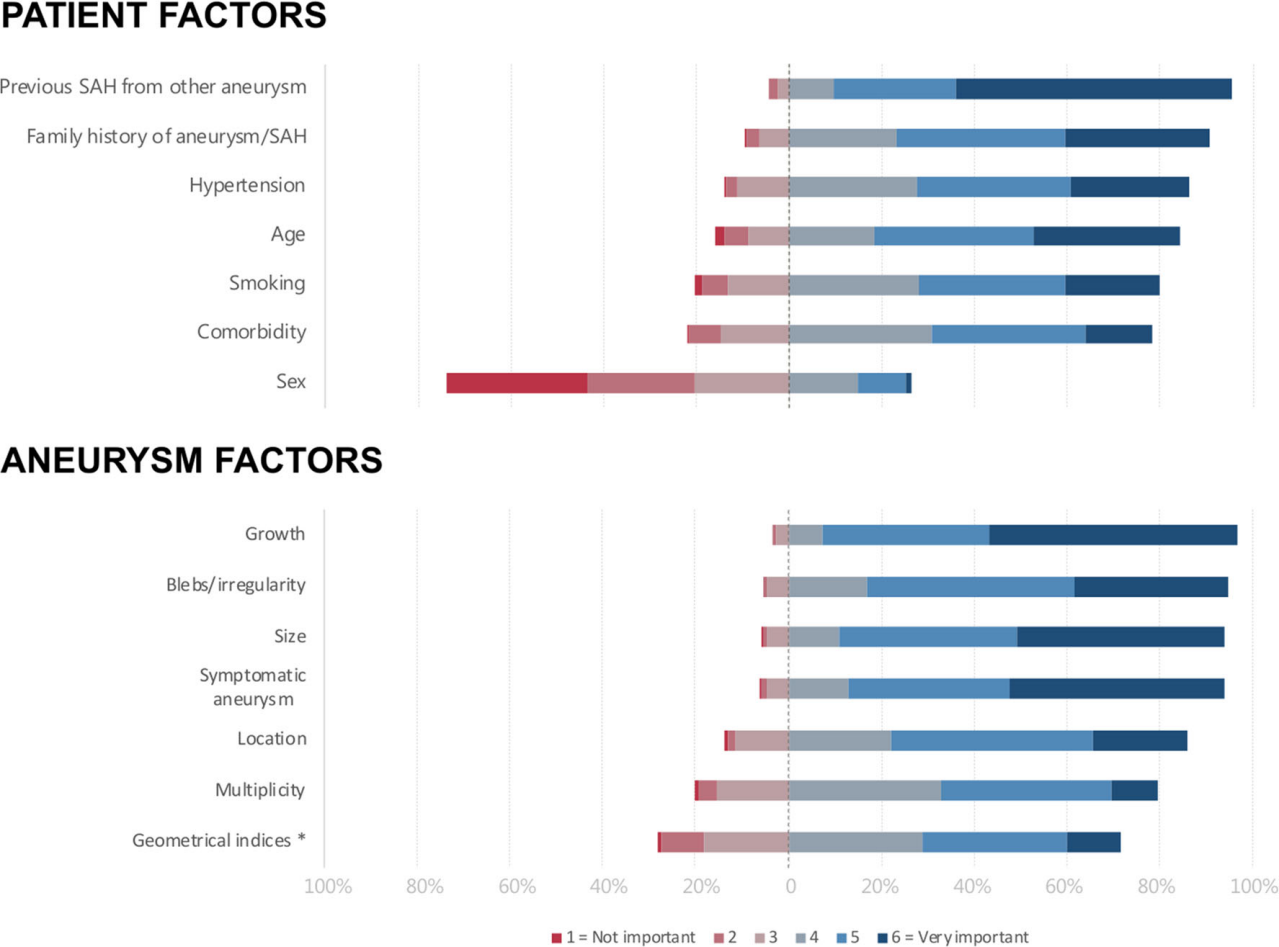
Visualization of how respondents rated the importance of patient and aneurysm factors when assessing risk of rupture. The asterisk is aspect ratio and similar indices

One hundred sixty respondents (40%) said their department used aneurysm size cut-off to guide treatment decisions, with a mean size 6 mm (SD, ± 2.4). Figure 2 visualizes the respondents’ rating of patient and aneurysm factors, respectively, in assessing aneurysm rupture risk. Previous subarachnoid hemorrhage from other aneurysms was rated as the most important patient factor, followed by age, and family history of aneurysm/subarachnoid hemorrhage. Sex was the only patient factor almost uniformly reported as unimportant. Aneurysm growth was rated as the most important aneurysm factor, followed by size, and symptomatic aneurysm.

### Case answers according to geographical region and gross domestic product per capita

Presented with a case of a 65-year-old male with an incidentally discovered 5-mm anterior communicating artery aneurysm, 87 of 351 respondents (25%) chose either “Observation with lifestyle changes” or “No further follow-up needed” whereas 264 (75%) advised intervention (endovascular or surgical prophylactic treatment). Figure 1 provides case details.

**Fig. 3:**
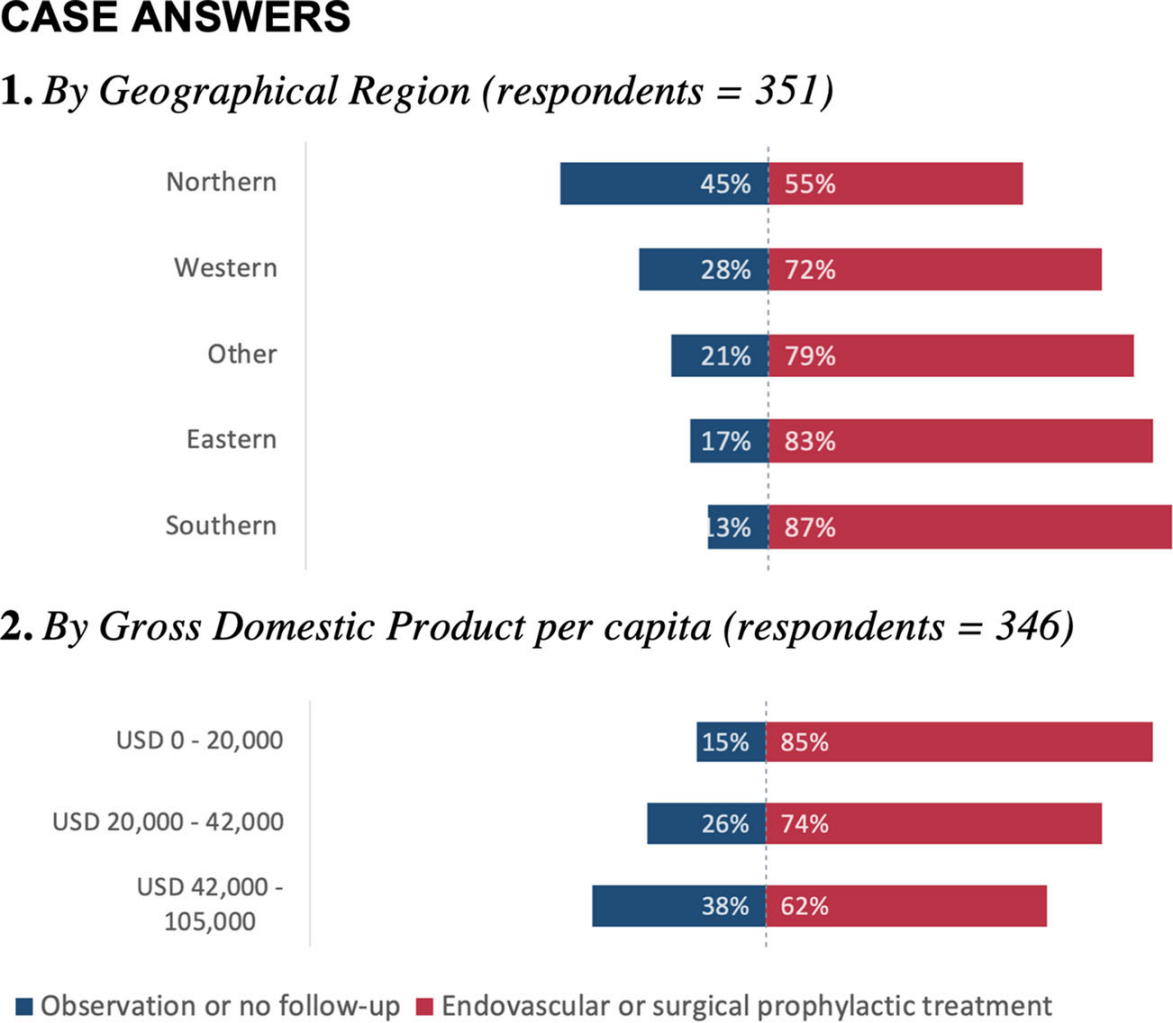
Case answers by geographical region and gross domestic product per capita

With regard to geographical region, 45% of the respondents from Northern Europe recommended observation or no follow-up compared with 13% from Southern Europe. The proportion of respondents recommending observation or no follow-up increased with the GDP per capita. These answers are visualized in Fig. 3, and absolute numbers of respondents are provided in Electronic supplementary material 2. Multinomial regression analysis of country categories and GDP per capita was statistically significant with a *p* value < 0.001 and an *R*^2^ of 0.53, suggesting that 53% of the variance in one of these two variables is explained by the variance in the other variable. Case answers and treatment volume were not statistically significantly associated (*p* = 0.52).

Twenty-one percent of respondents characterized themselves as vascular neurosurgeons. Forty-two percent of these recommended observation or no follow-up, compared with 21% among other respondents (*p* = 0.001). No statistically significant differences between vascular and non-vascular neurosurgeons was regarding the use of size cut-off (*p* = 0.83).

## Discussion

In this survey, more than half of the respondents reported that their department treated 0–25 unruptured aneurysms per year. Forty percent of respondents said their department used aneurysm size cut-off to guide treatment decisions. Presented with a case, respondents from countries with a lower GDP per capita recommended intervention more often than respondents from richer countries.

### Treatment volume

Guidelines from the American Heart Organization/American Stroke Organization recommends surgical treatment of unruptured aneurysms only in centers performing > 20 cases of unruptured aneurysms annually [4]. Thus, surprisingly many in our survey report a volume so low that treatment quality can be questioned. A Scandinavian study showed a trend towards lower 1-year mortality with higher treatment volume for unruptured intracranial aneurysms, but not for ruptured ones [12]. The authors hypothesized that surgical experience was more directly associated with outcome after treatment of unruptured aneurysms because these patients were otherwise healthy and underwent planned surgery [12]. As detailed in Electronic supplementary material 2, respondents from Southern and Eastern Europe reported lower treatment volumes. This finding warrants an open and thorough documentation of treatment and outcome.

### Aneurysm size cut-off and rating of risk factors

Almost half of the respondents reported that their department uses aneurysm size cut-off to guide treatment decisions, with a mean size of 6 mm. Surprisingly, this is smaller than the 7-mm cut-off often used in the literature [5, 13]. Though one of the most important risk factors for rupture, aneurysm size in itself is a parameter with low sensitivity and specificity for rupture [14, 15]. Relying too much on aneurysm size may thus cause overtreatment of large but benign aneurysms, and undertreatment of small but dangerous ones. In addition to patient factors, treatment decisions can be supported by aneurysm factors such as morphology and hemodynamics [2, 16–18], and magnetic resonance vessel wall imaging [19, 20].

Among patient-related risk factors, the respondents rated smoking as third lowest, and sex as least important. This is in stark contrast to previous literature, which has established that the most important risk factors for aneurysm rupture are smoking, hypertension, and female sex (especially postmenopausally), and that these three risk factors account for most of the variation in the incidence of aneurysmal SAH [21].

We do not know whether the respondents regard sex as an unimportant risk factor, or simply that they do not emphasize this risk factor when consulting individual patients. Similarly, we do not know how strictly the respondents use a size cut-off, and the survey question did not permit respondents to differentiate according to aneurysm location. Still, the respondents’ rating of risk factors and the large proportion of respondents that report using a cut-off size of 6 mm is an opportunity to encourage the neurosurgical community to ensure an evidence-based risk assessment, incorporating both aneurysm and patient-related factors [3, 4].

### Socioeconomic and regional differences

There was a clear association between a lower GDP per capita of respondents’ countries and a higher tendency to recommend intervention, when presented with the case of an incidentally discovered unruptured anterior communicating artery aneurysm. Treatment volume was not statistically significantly associated with different case answers. The proportion of respondents that said treatment decisions were taken by teams rather than individual doctors was higher in countries with middle and high GDP per capita. This leads to increased expenses by means of costly working hours of highly specialized personnel but hopefully also to better overall decisions and more efficient patient care pathways, incorporating a complex multifactorial assessment and less use of individual biases. As shown in Electronic supplementary material 2, respondents from countries with a higher GDP use magnetic resonance angiography and 3-dimensional digital subtraction angiography more often than respondents from countries with lower GDP per capita.

We are unaware of other questionnaire-based studies about the management of unruptured aneurysms except expert opinions [2], but clinical patient series support our findings of socioeconomic and geographical differences. A retrospective chart review of 424 consecutive patients with unruptured intracranial aneurysms managed in a single US center investigated factors associated with treatment (163 patients) versus observation (261 patients). As expected, younger patients, a lower Charlson Comorbidity Index, a larger aneurysm size and multiple aneurysms were associated with the decision to treat rather than to observe. Unexpectedly, a white race and living further from the medical center were also associated with a decision to treat [22]. Data from 57,000 unruptured intracranial aneurysms (18,000 treated) from the US National Inpatient Sample Database 2000–2010 showed that patients with private insurance coverage and white race were more likely to undergo treatment [23]. The external validity of these findings from the US health system (with a non-universal insurance system) on European health systems (with mostly government-funded or universal public insurance systems) is unknown [24].

### Vascular neurosurgeons

Twenty-one percent (*n* = 89) of respondents characterized themselves as vascular neurosurgeons, and the survey answers thus reflect opinions from doctors with varying experience with the decision-making in intracranial aneurysms. Presented with the case, the vascular neurosurgeons more commonly recommended observation. However, the proportion of vascular neurosurgeons was higher in the highest GDP tertile, and in Northern Europe (data not shown), and we cannot exclude confounding from these factors.

### Implications

We have found that the management of unruptured intracranial aneurysms is unequal across Europe. A self-reported survey, though prone to response bias, is a swift way to obtain data from many subjects and create an overview over an area of study. The response rate of 26% is good compared with many other surveys of doctors [7, 25], but we still consider it low. We are unable to verify the self-reported treatment volume with actual numbers of treated patients, and whether the actual treatment volume is sufficient for treatment and training quality. Therefore, the findings of questionably low treatment volumes and socioeconomic differences warrant further investigation. The survey detects a possible difference between the answers of vascular and non-vascular neurosurgeons, which needs further exploration. We will argue that all institutions treating intracranial aneurysms should report results openly to clinical quality registries, in order to aid comparisons and systematically drive improvements of the care quality across the entire region [26].

## Conclusions

According to answers in this self-reported survey, treatment of unruptured intracranial aneurysms in Europe is frequently performed in centers with low treatment volumes. Treatment decisions vary in relation to gross domestic product per capita and geographical regions. The treatment of unruptured intracranial aneurysms in Europe should be documented better, preferably by incorporating clinical quality registries.

## Electronic supplementary material

ESM 1 (PDF 680 kb).

ESM 2 (PDF 143 kb).

## Abbreviations

EANS: European Association of Neurosurgical Societies
GDP: Gross domestic product
PHASES: Population, hypertension, age, size of aneurysm, earlier SAH from another aneurysm, site of aneurysm
STROBE: Strengthening the reporting of observational Studies in epidemiology
UIATS: Unruptured intracranial aneurysm treatment score
