# Supplementary material for: Survey of European neurosurgeons’ management of unruptured intracranial aneurysms: inconsistent practice and organization
Source: Acta Neurochir (Wien). 2020 Sep 1;163(1):113–21. doi: 10.1007/s00701-020-04539-8 (PMC7778617; doi:10.1007/s00701-020-04539-8)
Supplement: Supplementary file 1 — (PDF 680 kb). [file 701_2020_4539_MOESM1_ESM.pdf]

Electronic supplementary material 1

Decision-making about unruptured intracranial aneurysms: a survey of European neurosurgeons.

Acta Neurochirurgica

Torbjørn Øygard Skodvin, Roar Kloster, Wilhelm Sorteberg, Jørgen Gjernes Isaksen

Corresponding author: Torbjørn Øygard Skodvin (University hospital of Nortern Norway, Tromsø, Norway; UiT The arctic university of Norway, Tromsø, Norway; Hospital of Southern Norway, Kristiansand, Norway).

E-mail: torbjorn.skodvin@gmail.com

Questionnaire **1**

UNRUPTURED INTRACRANIAL ANEURYSMS

SURVEY OF CURRENT MANAGEMENT

Introduction

This survey is part of an effort to increase knowledge about the discovery of and decisions about unruptured intracranial aneurysms, for the benefit of both patients and doctors. It takes about five minutes to complete.

You are contacted as one of a select individuals of European neurosurgeons. Your responses are highly valued, and needed to better understand the current controversies in this area. Your answers are completely confidential and will be released only as summaries in which no individual's answers can be identified.

The survey is a cooperation between all neurosurgical departments in Norway, led by researchers at UiT The Arctic University of Norway.

Thank you very much for helping with this important study.

UNRUPTURED INTRACRANIAL ANEURYSMS

SURVEY OF CURRENT MANAGEMENT

General information

*** 1. What is your age (years)?**

*** 2. What is your gender?**

- ◯ Female
- ◯ Male

*** 3. In what country do you currently reside?**

*** 4. Type of practice setting**

- ◯ University/teaching hospital
- ◯ General/regional hospital
- ◯ Private clinic/hospital
- ◯ Other (please specify)

*** 5. What is your position and role? (Please select all that apply.)**

- ☐ Head of department
- ☐ Consultant – vascular neurosurgeon
- ☐ Consultant – general neurosurgeon or other subspeciality
- ☐ One of the surgeons responsible for final treatment decisions about aneurysms
- ☐ Part of a vascular team responsible for final treatment decisions about aneurysms
- ☐ Other (please specify)

*** 6. In a typical month, how many patients with a newly discovered unruptured aneurysm are EVALUATED for treatment at your department?**

- ◯ < 1 (< 12 per year)
- ◯ 2-4 (13-48 per year)
- ◯ 5-15 (49-180 per year)
- ◯ 16-30 (181-360 per year)
- ◯ > 30 (> 360 per year)

*** 7. In a typical month, how many patients with an unruptured aneurysm are TREATED surgically/endovascularly at your department?**

- ◯ < 1 (< 12 per year)
- ◯ 1-2 (12-24 per year)
- ◯ 3-5 (25-60 per year)
- ◯ 6-10 (61-120 per year)
- ◯ > 10 (> 120 per year)

*** 8. Which procedures for prophylactic aneurysm treatment are performed at your department? (Please select all that apply.)**

- ☐ Surgical clipping
- ☐ Endovascular coil
- ☐ Endovascular stent/flow diversion
- ☐ Bypass operation
- ☐ Other (please specify)

9. Which procedures for prophylactic aneurysm treatment do you perform yourself? (Please select all that apply.)

- ☐ Surgical clipping
- ☐ Endovascular coil
- ☐ Endovascular stent/flow diversion
- ☐ Bypass operation
- ☐ Other (please specify)

Management routines

*** 10. From whom are patients with unruptured aneurysms referred to your department? (Please indicate the frequency of each alternative.)**

	Never	In special cases	Mainly	Always
General practitioner	○	○	○	○
Neurological department/clinic	○	○	○	○
Other neurosurgical department/clinic	○	○	○	○
Other non-neurosurgical or non-neurological department/clinic	○	○	○	○

Other (please specify)

____

*** 11. Who make final treatment decisions for patients unruptured aneurysms at your institution? (Please indicate the frequency of each alternative.)**

	Never	In special cases	Mainly	Always
Individual neurosurgeon	○	○	○	○
Individual neuroradiologist	○	○	○	○
Individual neurologist	○	○	○	○
Vascular team	○	○	○	○

Other (please specify)

____

*** 12. How many neurosurgeons are involved in the following phases of unruptured intracranial aneurysms evaluations at your department? (Please enter a number for each alternative.)**

Rupture risk assessment ____________________

Final treatment decisions ____________________

Surgical/endovascular treatment ____________________

13. ***This question concerns patients with newly discovered intracranial aneurysms that are admitted to the outpatient clinic at your department and subsequently receive prophylactic surgical/endovascular treatment.***

What is the normal waiting time for each of the following phases? (Please enter number of weeks for each alternative.)

From the department has received the referral and to the patient is seen in outpatient clinic?

[ ]

From the patient is seen in the outpatient clinic and to final treatment is performed?

[ ]

*** 14. Does your department use cut-off for aneurysm size to guide treatment decisions for unruptured aneurysms?**

- ◯ No
- ◯ Yes (please enter the cut-off size in millimetres.)

[ ]

*** 15. Please indicate the modality used to guide treatment decisions about unruptured aneurysms at your department.**

	Never	In special cases	Mainly	Always
Magnetic resonance angiography (MRA)	◯	◯	◯	◯
Computed tomography angiography (CTA)	◯	◯	◯	◯
2D digital subtraction angiography (2D DSA)	◯	◯	◯	◯
3D rotational angiography (3DRA / 3D DSA)	◯	◯	◯	◯

Risk assessment

*** 16. Assessment of rupture risk, *clinical factors*: Please indicate the importance of the factors below in assessing rupture risk of unruptured aneurysms.**

	Not important 1	2	3	4	5	Very important 6
Age	◯	◯	◯	◯	◯	◯
Sex	◯	◯	◯	◯	◯	◯
Previous SAH from other aneurysm	◯	◯	◯	◯	◯	◯
Family history of aneurysm/SAH	◯	◯	◯	◯	◯	◯
Smoking	◯	◯	◯	◯	◯	◯
Hypertension	◯	◯	◯	◯	◯	◯
Comorbidity	◯	◯	◯	◯	◯	◯

*** 17. Assessment of rupture risk, *aneurysm factors*: Please indicate the importance of the factors below in assessing rupture risk of unruptured aneurysms.**

	Not important 1	2	3	4	5	Most important 6
Size	◯	◯	◯	◯	◯	◯
Location	◯	◯	◯	◯	◯	◯
Blebs/irregularity	◯	◯	◯	◯	◯	◯
Symptomatic aneurysm	◯	◯	◯	◯	◯	◯
Multiplicity	◯	◯	◯	◯	◯	◯
Growth	◯	◯	◯	◯	◯	◯
Geometrical indices, as aspect ratio and others	◯	◯	◯	◯	◯	◯

*** 18. Patient-specific management: Please indicate the importance of the factors below with regard to management decisions for an individual patient with unruptured aneurysm(s).**

	Not important 1	2	3	4	5	Very important 6
Clinical risk factors †	◯	◯	◯	◯	◯	◯
Aneurysm risk factors ††	◯	◯	◯	◯	◯	◯
Internal department guidelines	◯	◯	◯	◯	◯	◯
Other colleagues' opinion or consensus	◯	◯	◯	◯	◯	◯
Your own expert opinion and overall clinical experience	◯	◯	◯	◯	◯	◯
The patient's wishes	◯	◯	◯	◯	◯	◯

† Age, sex, hypertension, smoking, comorbidity, family history, previous history of SAH, and more.

†† Size, location, blebs/irregularity, symptomatic aneurysm, multiplicity, growth, geometrical indices and more.

Cases

Please consider the following two cases.

Case 1: A patient with an unruptured intracranial aneurysm is referred to you. You receive a written referral, and must decide whether or not you also need to see the patient. Based on the supplied information about the patient and the aneurysm, you clearly see that surgical or endovascular treatment is not indicated.

*** 19. Question, case 1: What is your practice when informing about refusing treatment?**

	Never	In special cases	Mainly	Always
Neurosurgical outpatient consultation is not needed. The patient and/or referring doctor is notified per letter or phone call.	○	○	○	○
The patient is admitted for outpatient consultation.	○	○	○	○

Other (please specify)

Case 2: In your outpatient clinic, a 65-year old male presents with an anterior communicating artery (ACOM) aneurysm. The aneurysm has been incidentally discovered, and has a maximal diameter of 5 mm. The aneurysm wall is somewhat inhomogenous. The neuroradiologist informs you that endovascular treatment is possible.

The patient is diagnosed with hypertension, but does not smoke and is otherwise healthy. He is willing to undergo prophylactic treatment, but is not particularly anxious about the risk of rupture in the case of abstaining from prophylactic procedures.

Below is a CTA image of the aneurysm and surrounding vasculature.

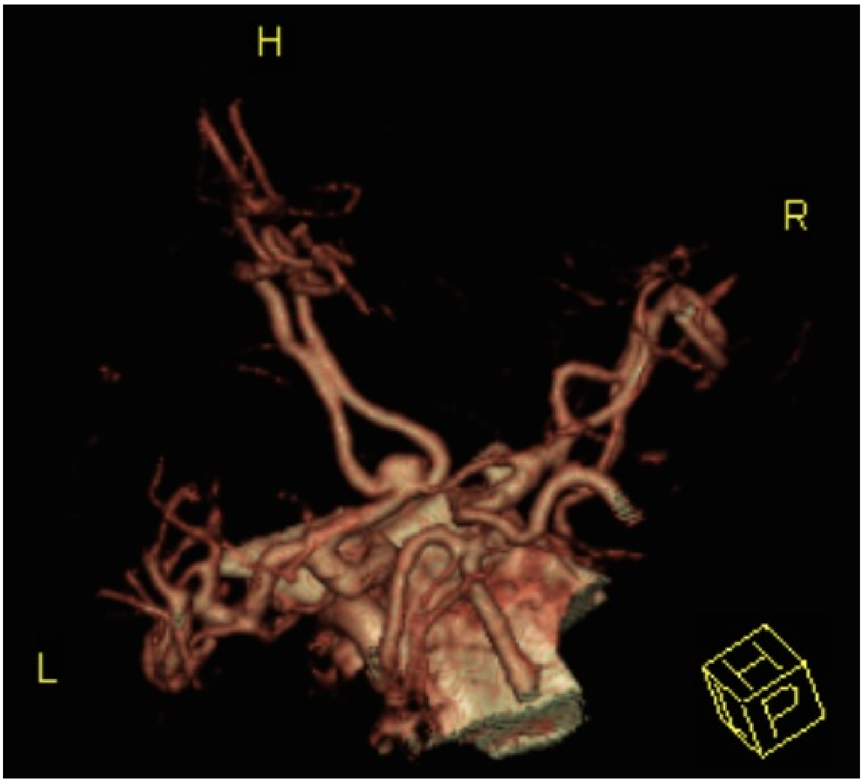

* **20. Question, case 2: Based on the supplied information, what is your most probable treatment decision for this patient?**

- ◯ Endovascular prophylactic treatment
- ◯ Surgical prophylactic treatment
- ◯ Observation with life-style changes
- ◯ No further follow-up needed

Thank you.

21. Is there anything you would like to add?

Thank you for your time. Your response is greatly appreciated.
