# Supplementary material for: Survey of European neurosurgeons’ management of unruptured intracranial aneurysms: inconsistent practice and organization
Source: Acta Neurochir (Wien). 2020 Sep 1;163(1):113–21. doi: 10.1007/s00701-020-04539-8 (PMC7778617; doi:10.1007/s00701-020-04539-8)

Electronic supplementary material 2

Decision-making about unruptured intracranial aneurysms: a survey of European neurosurgeons.

Acta Neurochirurgica

Torbjørn Øygard Skodvin, Roar Kloster, Wilhelm Sorteberg, Jørgen Gjernes Isaksen

Corresponding author: Torbjørn Øygard Skodvin (University hospital of Nortern Norway, Tromsø, Norway; UiT The arctic university of Norway, Tromsø, Norway; Hospital of Southern Norway, Kristiansand, Norway).

E-mail: torbjorn.skodvin@gmail.com

Supplemental tables of case answers and choice of diagnostic image modality.

TABLE I. Case answers according to GDP tertile* 1

TABLE II. Case answers according to geographical region* 2

TABLE III. Choice of diagnostic image modality according to GDP tertile* 3

TABLE IV. Choice of diagnostic image modality according to geographical region* 4

TABLE V. No. of respondents within each category of treatment volume, according to geographical region 6

FIGURE I. Percentage of respondents within each category of treatment volume, according to geographical region 6

TABLE I. Case answers according to GDP tertile*

Answer	GDP Tertile, no. (%)† Low	Intermediate	High	Total
GDP per capita in 10,000 USD, mean (SD)	1.23 (0.52)	3.71 (0.61)	6.26 (1.62)	346
All respondents	110 (100)	163 (100)	73 (100)	346
Observation or no further follow-up	16 (15)	42 (26)	28 (38)	86
Intervention (endovascular or surgical)	94 (85)	121 (74)	45 (62)	260

Abbreviations: GDP, Gross Domestic Product; SD, standard deviation.

* p value < 0.001 according to Chi squared test.

† Percentages within each tertile.

TABLE II. Case answers according to geographical region*

Answer	Northern	Western	Southern	Eastern	Other	Total
	Geographical area of Europe, no. (%)†					
GDP per capita of region in 10,000 USD, mean (±SD)	4.96 (1.39)	4.98 (1.54)	2.36 (0.76)	1.09 (0.45)	1.01 (0.20)	
All respondents	60 (100)	125 (100)	94 (100)	53 (100)	19	351
Observation or no further follow-up	27 (45)	35 (28)	12 (13)	9 (17)	4 (21)	87
Intervention (endovascular or surgical)	33 (55)	90 (72)	82 (87)	44 (83)	15 (79)	264

Abbreviations: GDP, Gross Domestic Product; SD, standard deviation.

* *p* value < 0.001 according to Chi squared test.

† Percentages within each region.

Northern region: Iceland, Denmark, Estonia, Faroe Islands, Finland, Guernsey, Iceland, Ireland, Isle of Man, Jersey, Latvia, Lithuania, Norway, Sark, Svalbard and Jan Mayen, Sweden, United Kingdom

Western region: Austria, Belgium, France, Germany, Liechtenstein, Luxembourg, Monaco, Netherlands, Switzerland

Southern region: Albania, Andorra, Bosnia and Herzegovina, Croatia, Gibraltar, Greece, Italy, Macedonia, Malta, Montenegro, Portugal, San Marino, Serbia, Slovenia, Spain, Vatican City.

Eastern region: Belarus, Bulgaria, Czech Republic, Hungary, Poland, Moldova, Romania, Russia, Slovakia, Ukraine.

Turkey, Israel were grouped into Other

TABLE III. Choice of diagnostic image modality according to GDP tertile*

Image modality	Frequence of use, no. (%)†		Total	Chart ‡
	Never/In special cases	Mainly/Always		
MRA				
GDP Tertile 1	90 (74)	32 (26)	122 (100)	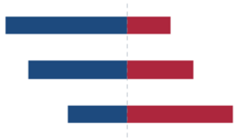
GDP Tertile 2	103 (60)	69 (40)	172 (100)	
GDP Tertile 3	28 (36)	50 (64)	78 (100)	
CTA				
GDP Tertile 1	31 (25)	91 (75)	122 (100)	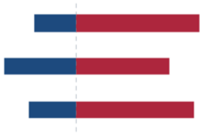
GDP Tertile 2	75 (44)	97 (56)	172 (100)	
GDP Tertile 3	22 (29)	55 (71)	77 (100)	
2D DSA				
GDP Tertile 1	65 (54)	57 (47)	122 (100)	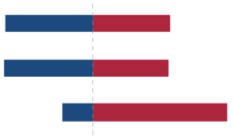
GDP Tertile 2	93 (54)	79 (46)	172 (100)	
GDP Tertile 3	46 (60)	31 (40)	77 (100)	
3D DSA				
GDP Tertile 1	69 (57)	53 (43)	122 (100)	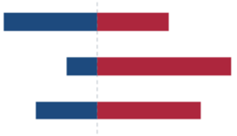
GDP Tertile 2	32 (19)	140 (81)	172 (100)	
GDP Tertile 3	29 (37)	49 (63)	78 (100)	

Abbreviations. 2D DSA, 2-dimensional digital subtraction angiography; 3D DSA, 3-dimensional digital subtraction angiography; CTA, computed tomography angiography; GDP, Gross Domestic Product; MRA, magnetic resonance angiography.

* GDP Tertile 1, 0-20,000 USD per capita; Tertile 2, 20,000-42,000 USD per capita; Tertile 3, >42,000 USD per capita.

† Percentages within each tertile.

‡ Charts over percentages. Blue indicates “never/in special cases”; red indicates “mainly/always”.

TABLE IV. Choice of diagnostic image modality according to geographical region*

	Frequence of use, no. (%)†			
Image modality	Never/In special cases	Mainly/Always	Total	Chart ‡
MRA				
Northern	31 (52)	29 (48)	60 (100)	
Western	62 (46)	73 (54)	135 (100)	
Other	15 (63)	9 (38)	24 (100)	
Eastern	43 (75)	14 (25)	57 (100)	
Southern	74 (73)	27 (27)	101 (100)	
CTA				
Northern	18 (31)	41 (69)	59 (100)	
Western	63 (47)	72 (53)	135 (100)	
Other	6 (25)	18 (75)	24 (100)	
Eastern	8 (14)	49 (86)	57 (100)	
Southern	35 (35)	66 (65)	101 (100)	
2D DSA				
Northern	32 (54)	27 (46)	59 (100)	
Western	77 (57)	58 (43)	135 (100)	
Other	9 (38)	15 (63)	24 (100)	
Eastern	36 (63)	21 (37)	57 (100)	
Southern	53 (52)	48 (48)	101 (100)	
3D DSA				
Northern	26 (43)	34 (57)	60 (100)	
Western	23 (17)	112 (83)	135 (100)	
Other	12 (50)	12 (50)	24 (100)	
Eastern	32 (56)	25 (44)	57 (100)	
Southern	39 (39)	62 (61)	101 (100)	

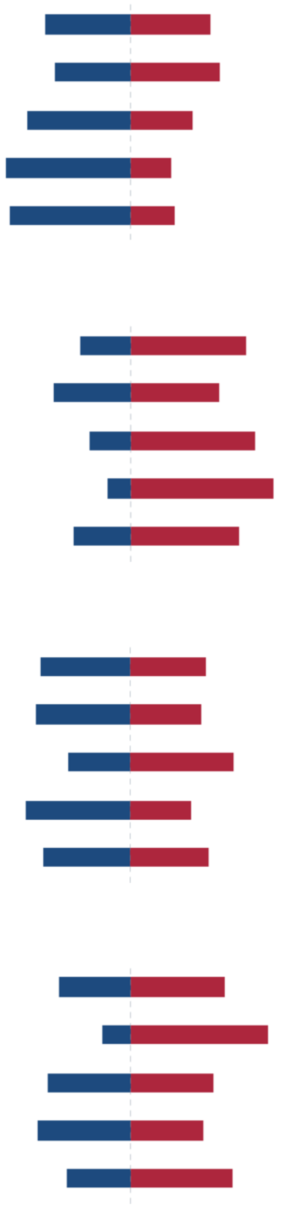

Abbreviations: 2D DSA, 2-dimensional digital subtraction angiography; 3D DSA, 3-dimensional digital subtraction angiography; CTA, computed tomography angiography; GDP, Gross Domestic Product; MRA, magnetic resonance angiography.

* GDP Tertile 1, 0-20,000 USD per capita; Tertile 2, 20,000-42,000 USD per capita; Tertile 3, >42,000 USD per capita.

† Percentages within each region.

‡ Charts over percentages. Blue indicates “never/in special cases”; red indicates “mainly/always”.
Northern region: Iceland, Denmark, Estonia, Faroe Islands, Finland, Guernsey, Iceland, Ireland, Isle of Man, Jersey, Latvia, Lithuania, Norway, Sark, Svalbard and Jan Mayen, Sweden, United Kingdom
Western region: Austria, Belgium, France, Germany, Liechtenstein, Luxembourg, Monaco, Netherlands, Switzerland
Southern region: Albania, Andorra, Bosnia and Herzegovina, Croatia, Gibraltar, Greece, Italy, Macedonia, Malta, Montenegro, Portugal, San Marino, Serbia, Slovenia, Spain, Vatican City.
Eastern region: Belarus, Bulgaria, Czech Republic, Hungary, Poland, Moldova, Romania, Russia, Slovakia, Ukraine.
Turkey, Israel were grouped into Other

TABLE V. No. of respondents within each category of treatment volume, according to geographical region

	Geographical area of Europe, no. (%)*					
No. of aneurysms	Northern	Western	Southern	Eastern	Other	Total
Total	6 (100)	144 (100)	116 (100)	65 (100)	30 (100)	420 (100)
< 12	6 (9)	21 (15)	38 (33)	19 (29)	13 (43)	97 (23)
12 - 24	22 (34)	36 (25)	39 (34)	22 (34)	10 (33)	129 (31)
25 - 60	16 (25)	58 (40)	28 (24)	10 (15)	6 (20)	118 (28)
61 - 120	15 (23)	24 (17)	9 (8)	10 (15)	1 (3)	59 (14)
> 120	6 (9)	5 (3)	2 (2)	4 (6)	0 (0)	17 (4)

* Percentages within each region.

Northern region: Iceland, Denmark, Estonia, Faroe Islands, Finland, Guernsey, Iceland, Ireland, Isle of Man, Jersey, Latvia, Lithuania, Norway, Sark, Svalbard and Jan Mayen, Sweden, United Kingdom

Western region: Austria, Belgium, France, Germany, Liechtenstein, Luxembourg, Monaco, Netherlands, Switzerland

Southern region: Albania, Andorra, Bosnia and Herzegovina, Croatia, Gibraltar, Greece, Italy, Macedonia, Malta, Montenegro, Portugal, San Marino, Serbia, Slovenia, Spain, Vatican City.

Eastern region: Belarus, Bulgaria, Czech Republic, Hungary, Poland, Moldova, Romania, Russia, Slovakia, Ukraine.

Turkey, Israel were grouped into Other

FIGURE I. Percentage of respondents within each category of treatment volume, according to geographical region

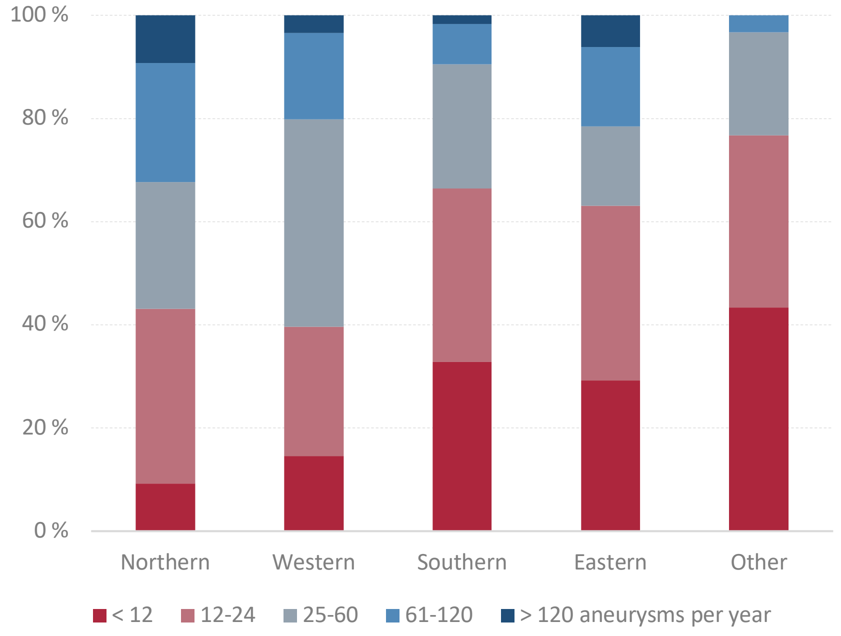

Supplement: Supplementary file 2 — (PDF 143 kb). [file 701_2020_4539_MOESM2_ESM.pdf]
